# Summation by Asian Elephants (*Elephas maximus)*

**DOI:** 10.3390/bs2020050

**Published:** 2012-03-27

**Authors:** Naoko Irie, Toshikazu Hasegawa

**Affiliations:** 1 Department of Evolutionary Studies of Biosystems, The Graduate University for Advanced Studies, 1560-3 Kamiyamaguchi, Hayama, Miura 240-0193, Japan; 2 Department of Life Sciences, Graduate School of Arts and Sciences, University of Tokyo, 3-8-1 Komaba, Meguro, Tokyo 153-8902, Japan; E-Mail: thase@darwin.c.u-tokyo.ac.jp

**Keywords:** numerical cognition, relative quantity judgment, summation, elephants

## Abstract

Recent empirical evidence for complex cognition in elephants suggests that greater attention to comparative studies between non-human primates and other animals is warranted. We have previously shown that elephants possess the ability to judge the difference between two discrete quantities, and unlike other animals, their choice does not appear to be affected by distance or overall quantity. In this study, we investigated Asian elephants’ ability to perform summation, as exemplified by the ability to combine four quantities into two sums and subsequently compare them. We presented two discrete sums (3–7) to the elephants by baiting two buckets; they were loaded sequentially with two discrete quantities (1–5 pieces) of food per bucket. All three elephants selected the larger grand sum significantly more often than the smaller grand sum. Moreover, their performance was not affected by either distance to the bait or the overall quantity evaluated. Studies report that the performance of other animal species on similar tasks declines as distance to the bait decreases and as the overall quantities evaluated increase. These results suggest that the numerical cognition of Asian elephants may be different from that of other animals, but further study is required to elucidate the differences precisely.

## 1. Introduction

This study investigated whether Asian elephants could perform summation in a relative quantity judgment (RQJ) task. This task requires an animal to make a dichotomous judgment: ordering unequal quantities by their magnitudes. Animals exhibit various types of numerical cognition, and one way to assess their abilities is via the RQJ task. Laboratory experiments have employed the RQJ task in the study of various species, including salamanders [1], mosquitofish [2], pigeons [3], squirrel monkeys [4,5], rhesus monkeys [6], gorillas [7], orangutans [8,9], and chimpanzees [10,11,12]. Previous studies indicate that animals’ RQJ performance varies with the disparity between the judged quantities, the magnitude of the stimuli, and the ratio between the stimuli subject to quantitative comparison. Disparity refers to the absolute difference between the quantities subject to comparison, while magnitude refers to the total quantity evaluated. The accuracy of RQJ tends to diminish as disparity decreases, magnitude increases, or the ratio between the quantities evaluated becomes more even. This can be explained by the accumulator model [13], which assumes that animals cannot count numbers but instead represent numerosity values as translated, cumulative quantities. Thus, errors committed by the animals also accumulate with increasing quantities, resulting in the decline of RQJ performance. 

Another model of numerical representation is called the object-file model [6]. This model states that animals encode separate items in terms of separate “object files,” which are limited in number to three or four, and these representations can be used for discrimination between numbers of quantities less than or equal to the number of the files. 

Some researchers have suggested that monkeys [14], fish [15] and human children [15] use some combination of these two distinct systems for representing numbers. Agrillo *et al.* reported that human undergraduate students and fish both performed equally well at an RQJ task involving small numbers (1–4), while their accuracy decreased with more even ratios between the quantities when the task involved large numbers (6–24 for human children and 4–16 for fish) [15].

We recently showed that Asian elephants also possess the ability to perform RQJ [16], but interestingly, the performance of elephants was not affected by disparity, magnitude, or the ratio of the comparisons, and their accuracy was stable throughout the task even when comparisons had numerosities larger than four (between quantities with magnitudes up to 6). Further study on numerical cognition in elephants is necessary to determine how their numerical representation is different from that of other animals.

Previous studies suggest that animals, including elephants, may perform RQJ in order to maximize the benefits associated with feeding. Numerical cognition may also play an important role for elephants in keeping track of numbers of other elephants as the size of a herd fluctuates between dry and rainy seasons. The size of the herd fluctuates according to the amount of food available at the time. African elephants form smaller groups during dry seasons than during wet seasons [17]. Elephants are known to discriminate between individuals accurately and to keep track of the members in the current group [18]. The use of numerical information, such as the number of individuals in the group, may help them to facilitate this process. Moreover, elephants form herds consisting of far larger numbers of individuals than the object-file representation range [17]. The ability to accurately assess a number of individuals above the object-file range may be beneficial for the elephants.

In order to determine how and in what ways elephants’ numerical competence is unique, we decided to investigate their RQJ performance under different conditions. In this study, elephants discriminated between groups of 3–7 objects. Moreover, we investigated whether elephants are able to employ the process of simple summation, which is thought to be a precursor to the formal addition of numbers in humans [19].

## 2. Methods

### 2.1. Ethics Statement

This work complies with American Psychological Association ethical statements and current Japanese animal welfare laws. The animals are located in the Kyoto Zoo (Kyoto, Japan), the Ueno Zoo (Tokyo, Japan), according to standards established by the Japanese Association of Zoos and Aquariums, and the Golden Triangle Asian Elephant Foundation (Chiang Rai, Thailand). The study was approved by the Kyoto Zoo, the Ueno Zoo, and the Golden Triangle Asian Elephant Foundation (GTAEF), and was carried out under the supervision of the elephants’ handlers.

### 2.2. Experimental Design

Three captive Asian elephants participated in the study. Their names are Mito, kept in the Kyoto Zoo (38-year-old female), Ashya, kept in the Ueno Zoo (30-year-old female), and Bo (33-year-old female), kept at GTAEF. All elephants were well trained to obey simple commands (*i.e.*, “come,” “go,” and “stay”). Ashya had previously participated in an RQJ experiment [15], but she had not been previously tested on summation. Mito and Bo had never been tested in any kind of numerical task before the current study. Moreover, the results of the experiment were not affected by possible learning effects, because we gave no feedback to the animals on whether or not they made a correct choice (elephants were rewarded for choosing both correct and incorrect buckets.) 

The experiment was carried out in Japan in 2007 and in Thailand in 2010. Two empty, opaque tin buckets, 1 m apart, were presented to the subject at a distance of 2 m. The experimenter dropped pieces of bait one by one into each bucket while elephants watched. The bait was added into each bucket in a “two-by-two process” in which the experimenter dropped a single quantity of bait into one bucket and then dropped another single quantity of bait into the other bucket. This was repeated until both buckets were filled with discrete total quantities. For example, if the comparison was 4 + 1 *vs.* 3 + 3, 4 pieces of bait were dropped into one bucket, then 3 pieces of bait were dropped into the second bucket, then 1 piece of bait was dropped into the first bucket, then 3 pieces of bait were dropped into the second bucket. Thus, the elephants had to add the quantities added to each bucket in order to assess how many pieces of bait were contained therein. Then, the elephants were given a “go” command and allowed to walk to one bucket and consume the bait inside. As soon as the elephant stood in front of one bucket, the unselected bucket was removed. Because the buckets were 50 cm deep, the elephants could not look into the buckets until they walked up to either bucket. Thus, they had no opportunity to visually compare the quantities in each bucket. 

The following summation comparisons were presented: 1 + 2 *vs.* 1 + 4, 2 + 1 *vs.* 1 + 4, 2 + 2 *vs.* 2 + 4, 1 + 3 *vs.* 5 + 1, 3 + 1 *vs.* 1 + 4, 4 + 1 *vs.* 3 + 3, 3 + 2 *vs.* 2 + 5, 3 + 2 *vs.* 5 + 2, and 5 + 1 *vs.* 3 + 4. Ashya and Bo were given additional probe comparisons: 2 + 3 *vs.* 5 + 2, 1 + 4 *vs.* 3 + 3, and 1 + 5 *vs.* 3 + 4. Probes are defined as comparisons in which the second quantity of the larger total sum was less than that of the smaller total sum (e.g., “1 + 3 *vs.* 5 + 1”, “3 + 2 *vs.* 5 + 2”). These probes were set to test whether or not the elephants avoided the pairs with the smaller second quantity. Unfortunately, Mito could not be tested with probe comparisons due to a husbandry issue that arose during the study.

Each comparison was presented six times, and presentation order was randomized. Pieces of the elephants’ favorite fruits were used as bait: pieces of orange for Mito, pieces of apple for Ashya, and pieces of cucumber for Bo. The bait pieces were cut into different sizes, so that total bait volume did not represent the number of bait pieces in each bucket. A visual comparison was made impossible by the position and depth of the buckets. We took this measure to ensure that the elephants did not visually compare the quantities, but rather counted the number of bait pieces as they were dropped into the buckets. In addition, the experimenter wore a cap and/or never looked up at the subjects during testing in order to avoid cuing the animals.

## 3. Results and Discussion

All subjects chose the bucket containing the larger number of bait pieces at significantly greater frequencies (binomial test: Mito: 68.5%; Ashya: 87%; Bo: 69.4%; p < 0.01) than the bucket containing the smaller sum. The performance under each condition is shown in Table 1. The elephants’ performance was not affected by disparity (Mito: *t*(7) = −0.88, *p* = 0.41; Ashya: *t*(7) = −0.68, *p* = 0.52, Bo: *t*(7) = −1.26, *p* = 0.25) or the total magnitude of the quantities in each comparison (Pearson’s correlation: Mito: *r* = 0.57, n = 9, *p* = 0.11; Ashya: *r* = 0.14, n = 9, *p* = 0.72; Bo: *r* = 0.31, n = 9, *p* = 0.42). However, Mito’s performance correlated with the total magnitude of the numbers compared: her accuracy increased when judging larger sums (Pearson’s correlation: *r* = 0.68, n = 9, *p* < 0.05).

**Table 1:** The presented summation comparisons and number of correct trials (out of six trials per comparison).

Sums	3 *vs.* 5	3 *vs.* 5	4 *vs.* 5	4 *vs.* 6	4 *vs.* 6	5 *vs.* 6	5 *vs.* 6	5 *vs.* 7	5 *vs.* 7	5 *vs.* 7	6 *vs.* 7	6 *vs.* 7
Formula	1 + 2 **	2 + 1	3 + 1	2 + 2 **	1 + 3 **	4 + 1	1 + 4	3 + 2 **	3 + 2 **	2 + 3 **	5 + 1 **	1 + 5 **
	*vs.*	*vs.*	*vs.*	*vs.*	*vs.*	*vs.*	*vs.*	*vs.*	*vs.*	*vs.*	*vs.*	*vs.*
	1 + 4	1 + 4	1 + 4	2 + 4	5 + 1	3 + 3	3 + 3	** 5 + 2	2 + 5	5 + 2	3 + 4	3 + 4
Mito	3	4	3	5	3	4	-	5	6	-	4	-
Ashya	6	5	4	5	5	6	4	5	6	5	5	6
Bo	5	4	3	3	5	3	4	5	5	3	5	5

**Figure 1:**
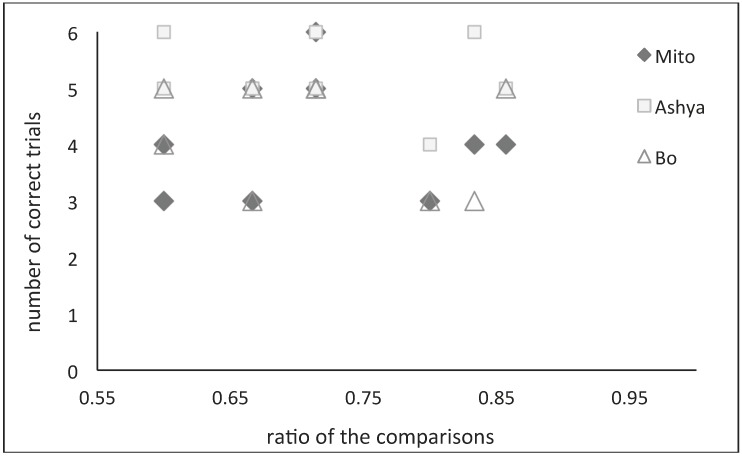
Ratios of comparisons and number of correct trials.

Accuracy was not affected by the ratio between the number of items in each bucket (*F*_(5,12)_ = 3.106, *p* = 0.200). Ratios were calculated by dividing the smaller sum in the comparison by the larger sum (e.g., for the comparison 1 + 2 *vs.* 1 + 4, the sums were 3 and 5; therefore, the ratio was 3/5 = 0.60). There were no significant correlations between the elephants’ performance and the ratio of the comparisons (Pearson’s correlation: Mito: *r* = 0.03, n = 9, *p* = 0.93; Ashya: *r* = −0.17, n = 9, *p* = 0.66; Bo: *r* = 0.25, n = 9, *p* = 0.52) (Figure 1). 

Ashya and Bo performed equally well on trials in which the probe comparisons were used (see Table 1). The percentage of correct responses for probe comparisons was 83.3% for Ashya and 73.3% for Bo (binomial test: *p* < 0.01); thus, Ashya’s and Bo’s levels of performance were not affected by the order in which quantities were presented. Mito’s accuracy rate for the comparisons 1 + 3 *vs.* 5 + 1 and 3 + 2 *vs.* 5 + 2 was 66.7%. Unfortunately, Mito could not be tested for the additional comparisons, and there were insufficient data to analyze her performance for a possible order effect.

## 4. Conclusions

The present findings indicate that Asian elephants are capable of performing summation and of mentally comparing successively presented sums. Ashya and Bo performed well for all comparisons regardless of disparity and magnitude. Mito performed better as the larger sum increased in magnitude. One possible explanation for Mito’s performance is that she was more motivated by comparisons involving larger sums because larger sums entailed a larger overall reward. 

Studies on other animals’ RQJ abilities suggested that their performance correlates closely with disparity and magnitude, indicating that their judgments rely on the approximate acquisition of quantities (*i.e.*, the accumulator model). In our previous study, no effects of disparity between, or magnitude of, the presented quantity pairs were seen in the performance of nine elephants. The results of the present study also indicated that elephants’ performance is not affected by disparities between the compared values or overall magnitudes larger than the object-file representation range. Therefore, our previous suggestion that the numerical representation of elephants may differ from those of other animals is supported by our present results.

The reason for the differences between the abilities of elephants and those of other animals in terms of quantity judgment and summation is still unclear. Nonetheless, the elephants’ judgment remained unaffected by the disparity or magnitude of the comparisons; this reflects faculties of mental summation and retention of numerical information and suggests that numerical cognition in elephants is complex (though incompletely understood). RQJ tasks can only indirectly investigate the numerical representations of animals; therefore, although we can conclude that elephants’ numerical representations may be different from those of other animals, we cannot determine how they differ. Therefore, we need to continue investigating their numerical competence using different tasks.
